# Supplementary material for: Level of and factors associated with optimal uptake of intermittent preventive treatment for malaria in pregnancy at private-not-for-profit health facilities in Kasese district
Source: PLOS Glob Public Health. 2024 Apr 3;4(4):e0002622. doi: 10.1371/journal.pgph.0002622 (PMC10990232; doi:10.1371/journal.pgph.0002622)
Supplement: Attachment — Submitted filename: PGPH-D-23-01526_commented script.pdf [file pgph.0002622.s002.pdf]

PLOS Global Public Health

Title: Level of and factors associated with optimal uptake of intermittent preventive treatment for malaria in pregnancy at private-not-for-profit health facilities in Kasese district

--Manuscript Draft--

Manuscript Number:	PGPH-D-23-01526
Article Type:	Research Article
Full Title:	Title: Level of and factors associated with optimal uptake of intermittent preventive treatment for malaria in pregnancy at private-not-for-profit health facilities in Kasese district
Short Title:	Level of and factors associated with optimal uptake of IPTp
Corresponding Author:	Julius Mutoro, Masters<br>Makerere University Faculty of Medicine: Makerere University College of Health Sciences<br>Kampala, UGANDA
Order of Authors:	Julius Mutoro, Masters
	Justus Barageine Kafunjo, Phd
	Freddy Eric Kitutu, Phd
	Joan N Kalyango, Phd
	Iving Mumbere
	Nathan Nshakira, Phd
Keywords:	Intermittent preventive Treatment (IPTp), Cotrimoxazole prophylaxis (CTXp), Private Non-profit Health facility, Malaria in pregnancy, sulfadoxine/pyrimethamine(SP) Knowledge on malaria, Optimal uptake, ≥3 doses of IPTp-SP or adherence scores ≥95% on CTX.
Abstract:	Abstract<br><br>Background: Malaria in pregnancy poses a high risk of poor maternal and neonatal outcomes and WHO recommends IPTp. However, its uptake has remained sub optimal among mothers who attend ANC at private not for profit health facilities.<br>Objectives: To determine the level and factors associated with uptake IPTp at private not for profit (PNFP) health facilities in Kasese District, Uganda.<br>Methods: A cross-sectional study involving 396 postpartum mothers in the postnatal wards of 8 PNFP health facilities in Kasese district was conducted in September 2022. One hospital and 2 HC IVs were purposively selected and 5 HC IIIs selected randomly. Mothers were consecutively selected and interviewer administered semi-Structured questionnaires were used to collect the data. Data were entered in epi-data version 3.1, cleaned and analyzed using STATA version 14. Data were adjusted for clustering & modified poison regression was used to determine associations of the factors and the outcome.<br>Results: Level of optimal uptake of IPTp was 51.5% (46.6-56.4). Being married (aPR=1.35, 95% CI=1.06-1.7, p=0.014), attending ANC more than 4 Visits (aPR=1.29, 95%CI=1.09-1.54, p<0.001), positively influence optimal uptake while not taking IPTp at recommended time intervals (aPR =0.49, 95%CI= 0.39-0.62 p<0.001) and mothers paying for IPTp drugs themselves (aPR =0.74, 95%CI=0.57-0.97, p=0.031) negatively influence optimal uptake. Conclusion: This moderate uptake of IPTp among pregnant mothers suggests insufficient protection of pregnant mothers against malaria. Efforts to improve ANC attendance, taking IPTp at recommended time intervals, not paying for ITPp drugs and marital status should be intensified.
Additional Information:	
Question	Response
Financial Disclosure	This research received no specific grant from any funding agency in the public,

Powered by Editorial Manager® and ProduXion Manager® from Aries Systems Corporation

Enter a financial disclosure statement that describes the sources of funding for the work included in this submission and the role the funder(s) played. This includes grants and any commercial funding of the work or authors.<br><br>This statement will be typeset if the manuscript is accepted for publication.<br><br>*Please review the submission guidelines and the instructions link below for detailed requirements and guidance.*	commercial or not-for-profit sectors.
**Competing Interests**<br><br>On behalf of all authors, disclose any competing interests that could be perceived to bias this work.<br><br>This statement will be typeset if the manuscript is accepted for publication.<br><br>*Please review the instructions link below and PLOS Global Public Health's competing interests policy to determine what information must be disclosed at submission.*	All authors declare that they have no competing interests
**Data Availability**<br><br>Before publication, Authors are required to make fully available and without restriction all data underlying their findings. Please see our PLOS Data Policy page for detailed information on this policy.<br><br>A **Data Availability Statement**, detailing where the data can be accessed, is required at first submission. Insert your Data Availability Statement in the box	Data set and study materials used during the study are available from corresponding author on reasonable request.

below.<br><br>Please see the data reporting section of our submission guidelines for instructions on what you need to include in your Data Availability Statement.<br><br>This statement will be typeset if the manuscript is accepted for publication.<br><br>*PLOS allows rare exemptions to address legal and ethical concerns. If you have legal or ethical restrictions, please use the box below to detail these in full sentences for the Journal team to consider.*	

Manuscript

Click here to access/download;Manuscript;Manuscript for_Mutoro julius.docx

Title: Level of and factors associated with optimal uptake of intermittent preventive treatment for malaria in pregnancy at private-not-for-profit health facilities in Kasese district

Authors

Mutoro Julius[1], Barageine Justus Kafunjo[1,2] Freddy Eric- Kitutu [1,3], Professor. Joan Kalyango [1,2]

Mumbere Iving [1,3], Nathan Nshakira [2,4]

Author affiliations

[1]Clinical Epidemiology Unit, College of Health Sciences, Makerere University

[2]Department of Obstetrics and Gynaecology, Makerere College of Health Sciences, Kampala, Uganda

[3] Department of Pharmacy, Makerere University School of Health Sciences, Kampala, Uganda.

[4] Department of community health, School of medicine, Kabale University Kampala Uganda

Corresponding author: Mutoro Julius. Email: Juliusmutoro@gmail.com (MJ)

Abstract

Background: Malaria in pregnancy poses a high risk of poor maternal and neonatal outcomes and WHO recommends IPTp. However, its uptake has remained ~~sub optimal~~ among mothers who attend ANC at private not for profit health facilities.

Objectives: To determine the level and factors associated with uptake IPTp at private not for profit (PNFP) health facilities in Kasese District, Uganda.

Methods: A cross-sectional study involving 396 postpartum mothers in the postnatal wards of 8 PNFP health facilities in Kasese district was conducted in September 2022. One hospital and 2 HC IVs were purposively selected and 5 HC IIIs selected randomly. Mothers were consecutively selected and interviewer administered semi-Structured questionnaires were used to collect the data. Data were entered in epi-data version 3.1, cleaned and analyzed using STATA version 14. Data were adjusted for clustering & modified poison regression was used to determine associations of the factors and the outcome.

Results: Level of optimal uptake of IPTp was 51.5% (46.6-56.4). Being married (aPR=1.35, 95% CI=1.06-1.7, p=0.014), attending ANC more than 4 Visits (aPR=1.29, 95%CI=1.09-1.54, p<0.001), positively influence optimal uptake while not taking IPTp at recommended time intervals (aPR =0.49, 95%CI= 0.39-0.62 p<0.001) and mothers paying for IPTp drugs themselves (aPR =0.74, 95%CI=0.57-0.97, p=0.031) negatively influence optimal uptake. **Conclusion:** This moderate uptake of IPTp among pregnant mothers suggests insufficient protection of pregnant mothers against malaria. Efforts to improve ANC attendance, taking IPTp at recommended time intervals, not paying for ITPp drugs and marital status should be intensified.

Key words:

Intermittent preventive Treatment (IPTp), Cotrimoxazole prophylaxis (CTXp), Private Non-profit Health facility, Malaria in pregnancy, sulfadoxine/pyrimethamine(SP) Knowledge on malaria, Optimal uptake, ≥3 doses of IPTp-SP or adherence scores ≥95% on CTX.

Background

Globally Malaria is a major public health problem with an estimated 219 million cases and 435,000 deaths, [1], Sub Saharan Africa continues having the highest incidences of malaria among the risky population that translates to 95% of the global burden of malaria cases [2]. In Uganda, malaria remains the highest

contributor of outpatient cases at 29.1% and 10.9 % of all deaths[3] and 69.7% positivity rate recorded at outpatient departments of five malaria reference centers across the country[4]. In high malaria transmission settings in Uganda, placental malaria has been reported at 44.6% among pregnant mothers. In Africa, malaria in pregnancy poses a 3-4 times risk of miscarriage, low birth weight and increased stillbirth[5]. In response, Uganda adopted the WHO recommendations[6] which call for daily co-trimoxazole for pregnant women who are HIV positive and 3 or more doses of IPTp-SP for pregnant women who are HIV negative. Although an analytical cross sectional study in Mbale Regional Referral Hospital among 184 mothers in labour linked optimal uptake of IPTp to reduction of malaria infections by 73.3% and anemia by more than half.[7], optimal uptake of IPTp is still quite low in Uganda at 14.7%[8]. There still exist inadequacies in knowledge of pregnant women on IPTp, side effects of the drugs and stock outs of IPTp supplies that limit optimal uptake in Uganda[9]. Though policies and guidelines are in place on IPTp, ~~they still have gaps in~~ ensuring adherence to optimal uptake of IPTp and this is evidenced by the insufficient monitoring mechanism of ensuring that mothers take the drug while away from the facility in the event that they did not take it as Directly observed treatment (DOT). In Kasese District, PNFPs are located in mountainous and densely populated areas and they constitute 11% (15/128) of the health facilities and offer ANC and maternity services to about 40% of the mothers in the communities. They charge some ANC care services like ANC cards and some investigations and this is likely to limit access of women with low incomes [10]. Numerous research on IPTp uptake have been conducted in public facilities, but they have not focused on pregnant women who use PNFP health facilities, which are the only closest facilities available to them in their ~~hard to reach~~ areas. If the situation persists, mothers may be more susceptible to malaria complications during pregnancy and the subsequent impacts on unborn babies. This justifies the need to determine the level and factors associated with optimal

uptake of intermittent preventive treatment (IPT) for malaria during pregnancy in private, non-profit health facilities in the Kasese district of Uganda.

The Ministry of Health will utilize the data to strengthen implementation of the policy and guidelines on the prevention of malaria. At facility level, it will enable health workers to identify important factors affecting IPTp and devise facility-based plans for improving the situation.

Methods

Study setting

This study was conducted at postnatal wards of 8 PNFP health facilities (1 hospital, 2 HCIV, 5 HCIIIs) in Kasese District which is predominantly rural and mountainous located approximately 350Km west of Kampala. The District borders with the Democratic Republic Congo (DRC) in the west and has 128 health facilities (4 hospitals, 5 Health centre (HC) IVs, 45 HC III, 74 HC II), among which 15 are PNFPs (1 hospital, 2 HC IV, 11 HCIII & 1 HCII). At the health facilities, the study was conducted in the postnatal wards of the selected health facilities because we expected mothers in postnatal ward to have completed the antenatal cycle. All the selected health facilities offer antenatal and postnatal care services and they have different working conditions and environments and these provide differential contexts for providing IPTp.

Study design, participant enrollment, and data collection

The study was a cross sectional study that included mothers in post-natal ward having delivered within 72 Hours, had attended ANC from any PNFP facility in the district and gave informed consent. Those who had delivered from other health facilities but were referred to the study site for special care of the infant or the mother. Purposive sampling for 3(hospitals &HCIV) and random sampling for 5 HCIIIs using the list of facilities from DHO's office ~~was done,~~ these facilities included: Kagando Hospital, St. Puals HCIV, Rwesande HCIV, Katadoba HCIII, Kasanga PHC HCIII, Kyarumba PHC HCIII, Nyabugando HCIII &

Kinyamaseka HCIII. Proportionate sampling was used to select the number of participants per health facility and consecutive sampling done for participants.

Data were collected from 396 participants using semi-structured questionnaires & verified using ANC cards by 8 trained research Assistants. These the questionnaires were translated into Lhukonzo as the commonly spoken language.

Sample size and power calculations

The sample size for the study was calculated using the formula for single proportions (modified Kish Leslie)[11] to cater for design effect. $N=Z^2\alpha/2\rho(1-\rho)/d2 *DE$. Where: N=396, Z is the standard normal value corresponding to 95% level of confidence which is 1.96, P is the percentage of mothers who took 3 or more doses of IPTp-SP, 14.7%,[8] q is 1-p, =1-0.147, d is the tolerable sampling error (precision) estimated at 5%, DE- is design effect, We multiplied by 2 to cater for the design effect. The minimum required sample size estimated was 386, However, we had collected extra 10 questionnaires and they were also added to make 396.

Statistical analysis

Data were transferred from the excel spread sheets into STATA version 14.0 for analysis and missing data were checked for each variable. Data was adjusted for clustering. To calculate the proportion of mothers who received optimal IPTp, we divided the number of participants who took three or more doses of IPTp by total number of eligible study participants. At univariate level, the data was presented as percentages and frequencies and categorical variables, medians with corresponding interquartile ranges and means and standard deviation for continuous variables. At Bivariate analysis, the association between the outcome (optimal uptake of IPTp) and the independent variables was done using modified Poisson regression reporting prevalence ratios (PRs). A bivariate model was run with the dependent variable (Uptake of three or more doses) with each of the independent variable each at a time to obtain un-adjusted prevalence ratios between the

outcome and each predictor. Independent variables with p-values less than 0.2 were selected for multivariate analysis. The selected variables from bivariate analysis ($p<0.2$) were then run in a multivariable model and then stepwise backward removal was performed to determine which factors remained significant at $p<0.05$. At this stage only 4 Variables remained significant in the model and then interaction terms were formed between them. A stepwise backward elimination method was then applied removing variables with the largest non-significant p values ($p>0.05$) systematically until only significant variables and those that improved the fit of the model were retained. The significant variables from the stepwise regression formed the crude model for the multivariable analysis to which confounding was assessed. A variable was considered to be a confounder when it changed the prevalence ratio by 10% or more and would be included in the final multivariable model. The prevalence ratios (PR) and 95% confidence intervals were presented as the measure of association between the different predictors and uptake of three or more doses.

Ethical considerations

The data collection for this cross sectional study was conducted between 8th sept and 23rd September 2022, Ethical approval was obtained from School of Medicine Research Ethics Committee (SOMREC) with the study number Mak-SOMREC-2022-398 and permission was also obtained from Clinical Epidemiology Unit (CEU) and from Kasese District Health Office and the respective health facilities where the study was conducted. Written informed consent were obtained from participants. Mothers below age of 18 years consented on assumption that they were emancipated minors. Though the participants consented to the study, they still had a right to discontinue their participation at any time of the study.

Results

Baseline characteristics

A total of 396 postpartum mothers from eight health facilities in Kasese district participated in the study between 8th sept and 23rd September 2022. During data collection, 10 more questionnaires to make 396 were collected and they were included in the analysis since we didn't want to lose information. Study participants had a mean (+/- SD) age of 26.1 (6.8) years and 269/396(67.9%) were married, about half 200/396 (50.5%) had primary as highest level of education and 272/396(68.7%) and resided in rural areas, 270/396 (68.2%) were un employed, 230/396(58.1%) live in a distance of less than 5 Km from the health facility and 305/396 (77.0%) lived with their husbands. Details of socio-demographics of the participants are shown in Table 1.

Table 1: Demographic characteristics of 396 mothers from 8 Health facilities in Kasese

Characteristic	Category	n(%)
Age	≤26 years	212(53.5)
	>26 years	184(46.5)
Marital status	Single	101(25.5)
	Married	**269(67.9)**
	Divorce	26(6.6)
	Widow	4(1.0)
Education level	No education	66(16.7)
	Primary	**200(50.5)**
	Secondary	105(26.5)
	Tertiary	25(6.3)
Residence	Rural	**272(68.7)**
	Urban	124(31.3)
Occupation of participants	Employed	32(8.1)
	Self employed	94(23.7)
	Unemployed	**270(68.2)**
Distance	Less than 5km	**230(58.1)**
	More than 5km	166(41.9)
Mothers living with their husbands	Yes	**305 (77.1)**
	No	91(22.9)

n-Number of participants, %-Percentage

Health seeking behaviors, IPTp drug factors and knowledge characteristics of respondents

~~Approximately a half~~ 211 (53.3%) of the mothers attended ≥4 visits, 250(63.1%) were escorted by their husbands, majority 281 (71.0%) amount paid per ANC visit, 203(57.2) took IPTp at recommended time intervals, one third 56(15.8) of the mothers met the costs for IPTp drugs, median parity was 2 and ranged from 2 to 4, majority for 306(77.3%) were multiparous, a third had a gestation age of 290(73.3%) at first ANC and inadequate knowledge and majority 300(84.5%)took IPTp-SP. From the analysis on the health seeking behaviors of IPTp drug and knowledge characteristics, Overall, 211/396 (53.3%) attended ANC care for less than 4 visits, 146/396 (36.9%) mothers were escorted by their husbands, 281/396 (71.0%) paid less than 10,000 for ANC services at the facilities, 203/355(57.2%) took IPTp at recommended time intervals. Majority (249/355, 70.1%) did not buy IPTp drugs. The median parity for mothers was 2 (range 2-4), In addition, 306/396 (77.3%) were multi-porous and more than a half 290/396(73.3%) mothers attended ANC during the 14-27 weeks of their gestation age, 281/396 (70.9%) had inadequate knowledge, 92.2%(365/396) of mothers were HIV-negative and 300/396 (84.5%) of the mothers took SP as shown in the table 2 below.

Table 1: Health seeking behaviors, IPTp drug and knowledge characteristics of mothers from 8 PNFP health facilities.

Characteristic	Category	N(%)
Number of ANC visits for mothers (N=396)	Less than 4	**211(53.3)**
	4 or more 4	185(46.7)
Mothers escorted by their husbands	Yes	146(36.9)
	No	**250(63.1)**
Amount paid per ANC visit (N=396)	<10,000	**281(71.0)**
	11,000-20,000	69(17.4)
	>20,000	46(11.6)
Taking IPTp at recommended time intervals (N=355)	Yes	**203(57.2)**
	No	152(42.8)
Who ~~met~~ costs for IPTp drugs(N=355)	Did not buy drugs	**249(70.1)**
	Self	56(15.8)
	husband/parent	50(14.1)
Parity(N=396)	Median(IQR)	2(2-4)
Gravidity(N=396)	Prime-parous	90(22.7)
	Multi-parous	**306(77.3)**
~~Gestation age~~ at ANC visit –weeks (N=396)	1-14	79(19.9)
	14-27	**290(73.3)**
	28-40	27(6.8)
Knowledge of mothers on Malaria in pregnancy(N=396)	Not adequate	**281(70.9)**
	Adequate	115(29.1)
HIV status	HIV Positive	31(7.8)
	HIV negative	365(92.2)
Type of IPTp Medicine taken	SP	**300(84.5)**
	Cotrimaxazole	20(5.6)
	Don't know	35(9.9)

N-Number of participants, %-Percentage

Level of optimal uptake of three or more doses of IPTp.

From the 396 ~~eligible~~ participants, 204 mothers took ≥3doses of IPTp-SP or ≥95% adherence to cotrimoxazole giving an overall prevalence of 51.5% (46.6-56.4). The prevalence varied across health facilities with the highest prevalence seen at Kagando Hospital (61.2%) and the lowest at Kyarumba PHC HCIII (33.3%)

Factors associated with uptake of three or more doses of IPTp.

At Bivariate analysis, 10 out of 20 variables were associated with optimal uptake of IPTp at p<0.2. they included: Marital status (CPR.=1.51 95%CI 1.15-1.99), occupation (CPR.=0.73 95%CI 0.53-0.99), and Distance from health facility (CPR.=084, 95%CI 0.0.68-1.02), number of ANC visits ≥4 (CPR.=1.45 95%CI 1.19-1.56), mothers escorted by husbands (CPR.=0.86, 95% CI 0.71-1.05), taking IPT as Directly observed therapy(DOT) (CPR.=0.69 95%CI 0.57-094), Taking IPTp at recommended time intervals (CPR.=0.49, 95%CI 0.38-0.62), malaria status of mothers in the last three months (CPR.=1.26 95%CI 0.99-0.1.61), mothers knowledge (CPR.=1.14 95%CI 0.93-1.39) as shown in table 2 and 3.

At multivariate analysis: four variables were found statistically significant at P<0.05 and they include: being married, (aPR 1.35, 95%CI 1.06-1.7, p=0.014), attending ANC more than 4 Visits, (aPR= 1.29, 95%CI=1.09-1.54, p=<0.001), IPTp drugs not taken at recommended time intervals, (aPR =0.49, 95%CI=0.39-0.62 p=<0.001), mothers paying for IPTp drugs themselves (aPR =0.74, 95%CI =0.57-0.97, p=<0.031). as shown in Table 3 and 4.

Table 3: Crude and adjusted analysis for demographic factors associated with uptake of IPTp among 396 mothers from 8 PNFP facilities in Kasese District.

			**Bivariate**		**Multivariate**	
**Variable**	**Optimal n(%) (N=204)**	**Sub optimal n(%) (N=192)**	**Crude PR 95% CI**	**p-value**	**Adjusted PR 95%CI**	**p-value**
**Age in years**						
Age in years ≤26	108(50.9)	104(49.1)				
>26	96(52.2)	88 (47.8)	1.02(0.84-1.24)	0.808		
**Marital status**						
Single	38 (37.6)	63(62.4)	Reference		Reference	
Married	153(56.9)	116(42.1)	1.51(1.15-1.99)	0.003	1.35(1.06-1.7)	0.014
Divorced	13(50.0)	13(50.0)	1.33(0.83-2.11)	0.225	1.39(0.9-2.15)	0.136
**Level of education**						
No education	35(53.1)	31(46.9)	Reference			
Primary	99(49.5)	101(50.5)	0.93(0.75-1.22)	0.612		
Post primary	70(53.8)	60(46.2)	1.02(0.77-1.34)	0.914		
**Residence**						
Rural	138(50.7)	134(49.3)	Reference			
Urban	66(53.2)	58(46.8)	1.05(0.86-1.29)	0.645		
**Occupation**						
Employed	22(68.7)	10(31.3)	Reference			
Self employed	47(50.0)	47(50.0)	0.73(0.53-0.99)	0.043		
Unemployed	135(50.0)	135(50.0)	0.73(0.56-0.95)	0.018		
**Distance from Health facility**						
Less than 5Km	127(55.2)	103(44.8)	Reference			
More than 5Km	77(46.4)	89(53.6)	0.84(0.68-1.02)	0.087		
**Live with Husband**						
Yes	159(52.1)	146(47.9)	Reference			
No	45(49.4)	46(50.6)	0.95(0.75-1.19)	0.66		

PR: Prevelence ratios, CI: Confidence interval, Pv-Probability value

Table 4: Crude and adjusted analysis for behavioral, reproductive and knowledge factors associated with uptake of IPTp among 396 mothers from 8 PNFP facilities in Kasese District.

			Bivariate		Multivariate	
**Variable**	**Optimal n(%) (N=204)**	**Sub optimal n(%) (N=192)**	**Crude PR 95% CI**	**p-value**	**Adjusted PR 95%CI**	**p-value**
**ANC Visits**						
Less than 4	90(42.6)	121(57.4)	Ref		Reference	
More than 4	185(61.6)	71(38.4)	1.45(1.19-1.56)	<0.001	1.29(1.09-1.54)	<0.001
**Mothers escorted by their husbands for ANC**						
Yes	82(56.2)	64(43.8)	Ref			
No	122(48.8)	128(51.2)	0.86(0.71-1.05)	0.152		
**Amount paid during ANC visit**						
<10,000	147(52.3)	134(47.7)	Ref			
11,000-20,000	34(49.3)	35(50.7)	0.94(0.72-1.23)	0.658		
>20,000	23(50.0)	23(50.0)	0.96(0.70-1.31)	0.775		
**Taking IPT as DOT(N=355)**						
Yes	171(59.2)	118(40.8)	Ref			
No	27(40.9)	39(59.1)	0.69(0.57-0.94)	0.019		
**Taking IPTp at recommended time intervals(N=355)**						
Yes	145(71.4)	58(28.6)	Ref		Reference	
No	53(34.9)	99(65.1)	0.49(0.38-0.62)	<0.001	0.49(0.39-0.62)	<0.001
**Meeting the costs for IPTp drugs(N=355)**						
Did not buy drugs	150(60.2)	99(39.8)	Ref		Reference	
Self	26(46.4)	30(53.6)	0.77(0.57-1.04)	0.09	0.74(0.57-0.97)	0.031
Husband/Parent	22(44.0)	28(56.0)	0.73(0.52-1.02)	0.06	0.79(0.58-1.06)	0.11
**HIV Status**						
HIV Positive	16(51.6)	15(48.4)	Ref			
HIV Negative	188(51.5)	177(48.5)	0.99(0.69-1.42)	0.991		
**Malaria status of mothers in last 3 months**						
Yes	45(43.3)	59(56.7)	Ref			
No	159(54.4)	133(45.6)	1.26(0.99-1.61)	0.065		
Parity						
Median(IQR) 2(1-4)			0.99(0.95-1.05)	0.842		
**Gravidity**						
Prime-parous	48(53.3)	42(46.7)	Reference			
~~Multi-porous~~	156(51.0)	150(49.0)	0.88(0.71-1.09)	0.25		
**~~Gestation age~~ at first Visit (weeks)**						

Less than 12 weeks	37(53.6)	32(46.4)	Reference	
12 or more weeks	167(51.1)	160(48.9)	0.12(0.75-1.22)	0.69
**Mother's age at first pregnancy**				
Median(IQR) 18(16-22)			0.99(0.97-1.00)	0.238
**Mothers' Knowledge on IPTp**				
Not adequate	139(49.5)	142(50.5)	Reference	
Adequate	65(56.5)	50(43.5)	1.14(0.93-1.39)	0.19

PR: Prevalence ratios, CI: Confidence interval, Pv-Probability value

Discussion

This cross sectional study was conducted in September 2022 among 396 post-partum mothers who had delivered within 72 hours at 8 private not for profit health facilities. The aim of this study was to determine the level and factors associated with uptake of Intermittent Preventive Treatment for malaria in pregnancy at private not for profit health facilities in Kasese district, Uganda.

Level of uptake of IPTp among mothers

Overall, a half of the mothers took optimal doses of IPTp with the least uptake recorded at Kyarumba PHC HCIII. This is far below the national target of 93% by 2020/2021, [12] but slightly higher than the 51% national IPTp+3 coverage for financial year 2020/21 as reported in the Annual Health sector performance report. [3]. These results indicate that only 6 in every 10 mothers are believed to be having some protection from malaria and with a reduced risk of getting complications during pregnancy. This is in line with the study in Malawi on the association between timing and number of antenatal care visits on uptake of IPTp that reported only 4 of every 10 mothers with low risk of malaria. [13]

The moderately high coverage of IPTp in our study could have been due to the strict implementation of Result Based Financing (RBF) strategy at health facilities where optimal uptake of IPTp is among the key indicators of focus. Under this strategy health services are subsidized and made affordable for the community members as evidenced in this report that 146/271(51.9%) of the mothers who took optimal dose of IPTp paid the least amount of 10,000 during their antenatal care visits at the PNFP facilities.

Additionally, interviewing mothers who had delivered within 72 hours might have led to this high results because this method reduced recall bias since some of them had attended ANC before, so it was easy for them to remember the drugs they had taken.

These results were consistent with a cross-sectional study in private health facilities in Tema metropolis in Ghana with relatively same sample size that reported 46.6% optimal uptake among mothers who had delivered. [14].

In Uganda, the available studies have been conducted in public health facilities and they have consistently reported low uptake of three or more IPTp doses evidenced by 14.7% in the study of determinants of uptake of malaria preventive interventions in eastern Uganda. [8]. The low uptake recorded in their study could have been due to missed opportunities as indicated in literature on the barriers of IPTp uptake in Uganda. [9]

Furthermore, the analysis of data from the Uganda Demographic and Health Survey (2016) on uptake of IPTp revealed coverage of 18% optimal doses of IPTp [15] lower than 41% reported in the Uganda malaria indicator survey [16]. Besides, this may indicate geographical variations in the intervention uptake across the country. Different study methodologies could also explain the difference. For example, the survey sampled respondents from the entire country while this study drew its sample from only one district.

Demographic factors associated with optimal uptake of IPTp for mothers

From this study, being married was significantly associated with optimal uptake of IPTp suggesting that married mothers had 35% higher chances of completing optimal doses. This may be explained by the emotional and financial support from their husbands as well as reminders to keep appointments. [17]. It could also be that single or divorced women get traumatized and stigmatized for getting pregnant out of matrimony and end up not taking IPTp hence a need for their social protection. Results from the six

districts of Tanzania has also indicated married women as more likely to take optimal doses of IPTp compared to the single women. [18]. Some studies have shown a positive association between presence of husband-wife discussion about health matters, such as family planning and utilization of various health services[19].

Contrary to our study, a cross sectional study of 239 respondents in Northern Nigeria reported mother's advanced age positively influencing optimal uptake of IPTp [20]. This could be explained by the fact that old mothers are expected to have vast experiences and knowledge on pregnancy related issues, this is likely to increase the uptake. Nonetheless, adolescent pregnant girls are less likely to attend an ANC and seek timely healthcare due to stigmatization and financial constraints.

Although level of education was not significantly associated with optimal uptake of IPTp in this study, Having secondary or higher level of education was associated with optimal uptake of IPTp among mothers in a study of prevention of malaria among women of reproductive age in Mozambique , [21]. However, significant association was reported in Arusha among a slightly higher sample size of post-delivery mothers. [22]. These variations may be explained by 50.2% and 16.7% of our mothers having primary and no education respectively as the highest level of education and reside in the remote areas of the district.

Like the cross sectional study in Mali among 2382 mothers, [23], our study also ~~didn't report~~ any significant association between residence and uptake of IPTp. This could be due to women in urban areas being not health conscious during their pregnancy and the quality of antenatal care services received by them before delivery. This is was also expressed in recommendations of a cross sectional study in Tanzania that aimed at curbing geographical barriers in uptake of IPTp[18],

Our study didn't find a significant association of occupation with optimal uptake of IPTp. However, in Ogbomoso, Oyo State, Nigeria, study results revealed an association of being employed with uptake of

Optimal IPTp contrary to our study finding [24]. This difference could be due to their large sample size and their inclusion criteria was not strict, it included both pregnant and post-partum mother.

Furthermore, distance from the health facility not been associated with uptake of IPTp, Across sectional study of 307 mothers conducted in Burundi reported a significant difference in the uptake of IPTp by mothers who stay less than 5 km from the health facility [25]. This difference could have arisen because they used community based approach of reaching the mothers in the settlements and yet the current study enrolled mothers at the health facility.

The mothers who live with their husbands were more likely to take optimal doses of IPTp as reported among postpartum women in Tandahimba district, Tanzania[26], However, in our study this had no significant association on the uptake of IPTp.

Behavioral factors associated with optimal uptake of IPTp

Additionally, we found that number of ANC visits was significantly associated with optimal uptake of IPTp dose where mothers who attended ≥4 visits were 29% more likely to take optimal IPTp versus mothers who attended less than 4 visits. Hence the need for motivation strategies for mothers to attend more ANC visits. This finding is in consonance with reports among mothers at a university Hospital, Kumasi-Ghana in 2018 which indicated that the number of ANC visits positively influencing the recommended IPTp uptake [27]. These results are ~~consistent to~~ the aOR = 1.55 (95% CI = 1.34–1.80) reported in a similar cross sectional study in Malawi though they interviewed mothers with live birth in the 2 years preceding the survey of 2015 to 2016 Malawi Demographic and Health Survey. [28] . Nonetheless the high ANC visits does not translate to optimal uptake of IPTp [29]

As reported in this study, it was also found that DOT practice, and health talk at the facility confirmed by ANC clients as satisfactory did not positively influence uptake of IPTp [30]. However, a cross sectional

study of pregnant women attending a tertiary hospital in north eastern Nigeria reported high odds of 4.6 for completing optimal doses among mothers who adhere to DOT compared to those who didn't adhered to DOT.[31]. The sample size of 184 was quite small and the study was conducted in a tertiary hospital in the town, Therefore, there is a likelihood of getting mothers who are knowledgeable and responsive to their lives compared to our study that was conducted in the remote areas of the district.

The study further observed that taking IPTp drugs at the recommended time intervals was significantly associated with optimal uptake of IPTp. Mothers who did not take IPTp at the recommended intervals had 51% less likely to complete optimal doses. The irregular attendances make mothers skip appointments hence fail to complete their doses. System and social challenges like lack of transport might have limited the mothers from keeping appointments as well as stock outs hence failing to adhere to the stipulated time intervals for taking drugs [32].

Compared with other studies, this study further demonstrated a significant association between mothers who bought IPTp drugs for themselves and optimal IPTp uptake indicating 26% less chances of completing optimal doses compared to those that did pay for IPTp drugs. This implies that payment for IPTp drugs by mothers negatively affects uptake of optimal doses. This is consistent with a qualitative study finding in the eastern and west Nile regions that reported mothers paying $0.28 for IPTp drugs at the health facility. [33]. This significant association can be due to that fact that when mothers buy drugs for themselves, they attach more value to the drugs and hence increasing uptake. In another qualitative study on perceived costs and barriers on IPTp uptake in Mali, mothers attested to paying varying amounts for IPTp drugs at different health facilities. [34]. In our study majority 134(47.7) of our mothers also paid less than $ 2.78 to access ANC services.

Contrary to our finding, a cross sectional study of 556 mothers from 9 selected health facilities in Arusha found early initiation of ANC being associated with IPTp uptake. [22]. In their analysis clustering was not catered for and yet they collected data from 9 health facilities and this is likely to introduce type 1 error in their results. ANC attendance alone is unlikely to enhance the uptake of this intervention. In north Tanzania, the results of a cross sectional study has also revealed that ANC timing does not improve uptake of IPTp as it would be expected[28].

In regard to the HIV status of mothers, a cross-sectional study of 353 mothers in Tanzania reported adherence to CPT of above among mothers and its significant association with IPTp uptake [35]. However, our study did not find a significant association between HIV status and uptake of optimal IPTp. This is mostly due to smaller sample size in our study especially for HIV positives.

Despite having a high proportion 26.3% of mothers with malaria episodes in the last 3 mothers, the findings revealed no significant association with optimal uptake of IPTp contrary to the contrary to the cross sectional study in Nigeria [36]. This indicates that suffering from malaria did not influence the uptake of the malaria preventive measures in pregnancy.

Payment of user fees to access antenatal care has been pointed out as a key factor associated with optimal utilization of ANC services in a five-country analysis of national service provision assessment surveys [37] though it was not found significant in our study. However, majority (71.0%) of the respondents spent $ 3 on each visit for ANC services. This could have also limited uptake of the services. Additionally, a study in Mali, has recommended removal of user fees for ANC and child birth services to increase their uptake.[34].

In our study, only 36.9% of the husbands escorted their wives to health facilities for ANC and this was not found to significantly influence uptake of IPTp as reported in Kisungu HCIII [38]. This might have been due to the low power in our study to detect the detect the significant difference.

As observed among postpartum women in Tanzania's Tandahimba district [26], mothers who live with their husbands are more likely to take the recommended doses of IPTp. However, in our study, there was no evidence of a significant correlation between this and IPTp uptake. However, in our study this had no significant association on the uptake of IPTp.

Knowledge and reproductive factors associated with optimal uptake of IPTp

The current study did not find an association with knowledge of IPTp contrary to this other study which reported an increased likelihood of uptake of IPT as significantly associated with knowledge of 400 post-partum mothers in Cameroon among [39]. Similarly a study in Mali also reported significant association[40], The current study being conducted in only private not for profit health facilities might have contributed to this none coherence findings with other studies.

Like in the cross sectional study in western Kenya in 2020, parity was not found to influence uptake of optimal IPTp. [41]. This is contrary to the results from a cross sectional study in Sierra Leone among 8526 that reported higher parity (>4) being associated with lower odds of taking IPTp-SP.[42].

In a rural to urban prospective cohort study in Mali, positive association was reported among prime gravida than in multi-gravid, contrary to our study. [43]. This could probably be due to the high proportion of prime-gravid mothers (56%) in their study compared 22.7% in the current study. Additionally, it has been found that primagravidae, or first-time mothers, are more susceptible to malarial infection than second- or third-time mothers (multigravidae). It is because of the development of placental parasite-

specific immunity during the second and third pregnancies that multigravidae are resistant to malarial infection. [44]

Our study had no significant association of mothers living with their husbands on uptake of IPTp. however, a study of among postpartum women in Tandahimba district, Tanzania reported a significant association [26].

Strength and limitations of the study

Self-reported adherence to uptake of IPTp especially for mothers who bought IPTp drugs from private clinics and took them from home was not possible to truly ascertain whether they took the recommended doses, this might have introduced reporting bias hence over estimation of the outcome. Since the study was targeting post-partum mothers, some might have not recalled the drugs they took during their ANC visits leading to bias, However, this was minimized by showing samples of SP or cotrimoxazole to them in order for them to relate their responses to the drug in question. Interviewing mothers who had delivered within 72 hours might have led to these high level of optimal uptake that was reported because this might have increased recall bias. No causal inferences may be drawn from these findings because of snapshot nature of cross-sectional studies.

Conclusion

The study has established that about a half of the pregnant mothers completed ≥3 doses of IPTp-SP or ≥ 95% adherence to cotrimoxazole in private not for profit health facilities in Kasese. However, this is substantially lower than the national expected target of 80%. Based on the research results, being married, attending more than 4 ANC visits, taking IPTp drugs at recommended intervals and mothers buying IPTp drugs by themselves were significantly associated with optimal uptake of IPTp. Given the location of the PNFP facilities in the underserved remote areas in the district, focusing on increasing early initiation of

ANC, health education on malaria in pregnancy and IPTp as well as removing charges on ANC services may therefore be important in increasing the uptake of IPTp in this district.

Recommendations:

Ministry of Health (MOH) should fully implement the Public Private Partnership for Health policy guidelines and set uniformly subsidized and affordable costs for essential maternal services, DHO’s office to keep track of the IPTp supplies and re-allocate drugs from over stocked to under stocked health facilities, DHO’s, MOH to orient the health workers on Goal oriented antenatal and health workers to sensitize the mothers on the prevention and control of malaria in pregnancy including the importance of antenatal care services. However, since this study focused on the client’s perspectives further studies are needed to examine the facility and provider’s factors that might contribute to promotion of uptake.

Acknowledgement

We thank all the people who were involved in the synthesis of this research up to its completion, and grateful to Kasese district health officer for the administrative support, study participants for consenting to participate in this study, the health facility in charges and research assistants for their involvement in this study, as well as the staff and students in the Clinical Epidemiology Unit, Makerere University.

List of abbreviations

ANC: Antenatal Care

ART: Antiretroviral Therapy

CMHS: College of Medicine and Health Sciences

CPT: Cotrimoxazole Preventive Therapy

CTXp Cotrimoxazole Prophylaxis

DHO: District Health Officer

DOT: Directly Observed Therapy

DRC: Democratic Republic of Congo

HC III: Health Centre III

HC IV: Health Centre IV

HSD: Health Sub-District

IPTp: Intermittent Preventive Treatment of malaria in pregnancy

IPTp-SP: Intermittent preventive Treatment for malaria in pregnancy with sulfadoxine/pyrimethamine

MIP: Malaria in Pregnancy

MoH: Ministry of Health

PNC: Postnatal Care

PNFP: Private Not for Profit

SOMREC: School of Medicine Research Ethics Committee

WHO: World Health Organization

Declarations

I Mutoro Julius declare that this dissertation titled "Level and Factors Associated with Optimal Uptake of Intermittent Preventive Treatment for Malaria in Pregnancy at Private Not for Profit Health Facilities in Kasese District" is my original work and has not been presented for any other award in any other University or training institution.

Consent for publication

Not applicable

Data availability statement

Data set and study materials used during the study are available from corresponding author on reasonable request.

Competing interests: All authors declare that they have no competing interests

Funding

This research received no specific grant from any funding agency in the public, commercial or not-for-profit sectors.

Disclaimer:

The content of this report is solely the responsibility of the authors and does not necessarily represent the official views of Makerere University.

Conflict of interest

All authors declare no conflict of interest.

Author's contributions

Mutoro Julius: Contributed to study conceptualization, Visualization, Investigation, Methodology, Project administration, Data collection and analysis, Resources, Software, writing – original draft, Writing – review & editing.

Barageine Justus Kafunjo: Contributed to study conceptualization, Methodology, Supervision, Validation, Writing – review & editing, interpretation of data

Freddy Eric-Kitutu: Contributed to study conceptualization, revision and writing of the protocol and manuscript – reviewing & editing

Joan Kalyango: Contributed to study design, conceptualization supervision and revision and conduct of the study.

Mumbere Iving: Contributed to study conceptualization, analysis and writing

Nathan Nshakira: Contributed to drafting the manuscript

All authors participated in writing of the manuscript, and approved the final manuscript.

References

1. WHO, *World Malaria Report 2018*. 2018: Geneva.
2. WHO, *WHO guidelines for Malaria, feb 2021*, in *Preventive chemotherapies & Mass drug administration*. 2021, WHO: Geneva. p. 9.
3. MOH, A., *Annual Health sector perfomance report* 2021: Kampala p. 20.
4. Okiring, J., et al., *Relationships between test positivity rate, total laboratory confirmed cases of malaria, and malaria incidence in high burden settings of Uganda: an ecological analysis*. Malar J, 2021. **20**(1): p. 42.
5. Saito, M., et al., *Deleterious effects of malaria in pregnancy on the developing fetus: a review on prevention and treatment with antimalarial drugs*. Lancet Child Adolesc Health, 2020. **4**(10): p. 761-774.
6. WHO, *WHO policy brief for the implementation of intermittent preventive treatment of malaria in pregnancy using sulfadoxine-pyrimethamine (IPTp-SP)*, WHO, Editor. 2013, WHO.
7. Nekaka, R., et al., *Malaria preventive practices and delivery outcomes: A cross-sectional study of parturient women in a tertiary hospital in Eastern Uganda*. PLoS One, 2020. **15**(8): p. e0237407.
8. Wafula, S.T., et al., *Determinants of uptake of malaria preventive interventions among pregnant women in eastern Uganda*. Malar J, 2021. **20**(1): p. 5.
9. Muhumuza, E., et al., *Factors associated with use of malaria control interventions by pregnant women in Buwunga subcounty, Bugiri District*. Malaria journal, 2016. **15**(1): p. 1-8.
10. DHO, *Kasese District Annual performance Report* 2021. p. 12.
11. Pourhoseingholi, M.A., M. Vahedi, and M. Rahimzadeh, *Sample size calculation in medical studies*. Gastroenterol Hepatol Bed Bench, 2013. **6**(1): p. 14-7.

12. MOH, *The Uganda Malaria reduction strategic plan 2014-2020*, MOH, Editor. 2014, MoH: Kampala p. 29.
13. Nkoka, O., T.W. Chuang, and Y.H. Chen, *Association between timing and number of antenatal care visits on uptake of intermittent preventive treatment for malaria during pregnancy among Malawian women.* Malar J, 2018. **17**(1): p. 211.
14. Amankwah, S. and F. Anto, *Factors Associated with Uptake of Intermittent Preventive Treatment of Malaria in Pregnancy: A Cross-Sectional Study in Private Health Facilities in Tema Metropolis, Ghana.* J Trop Med, 2019. **2019**: p. 9278432.
15. Okethwangu, D., et al., *Factors associated with uptake of optimal doses of intermittent preventive treatment for malaria among pregnant women in Uganda: analysis of data from the Uganda Demographic and Health Survey, 2016.* Malaria Journal, 2019. **18**(1): p. 250.
16. MNCD, *Uganda Malaria Indicator Survey 2018-19. Kampala, Uganda,*, U.B.o.S.U.a.I. National malaria control Division (UNMCD, Editor. 2018, MOH: Kampala p. 23.
17. Mutanyi, J.A., et al., *Determinants of the uptake of intermittent preventive treatment of malaria in pregnancy with sulphadoxine pyrimethamine in Sabatia Sub County, Western Kenya.* Infect Dis Poverty, 2021. **10**(1): p. 106.
18. Exavery, A., et al., *Factors affecting uptake of optimal doses of sulphadoxine-pyrimethamine for intermittent preventive treatment of malaria in pregnancy in six districts of Tanzania.* Malaria Journal, 2014. **13**(1): p. 22.
19. Kamal, S.M. and M.A. Islam, *Interspousal communication on family planning and its effect on contraceptive adoption in Bangladesh.* Asia Pacific Journal of Public Health, 2012. **24**(3): p. 506-521.
20. Iliyasu, Z., et al., *Adherence to intermittent preventive treatment for malaria in pregnancy in urban Kano, northern Nigeria.* Pathogens and Global Health, 2012. **106**(6): p. 323-329.
21. Afai, G., et al., *Factors associated with knowledge about malaria prevention among women of reproductive age, Tete Province, Mozambique, 2019-2020.* Malar J, 2022. **21**(1): p. 76.
22. McHwampaka, W.M., et al., *Factors affecting uptake of≥3 doses of Sulfadoxine-Pyrimethamine for malaria prevention in pregnancy in selected health facilities, Arusha region, Tanzania.* BMC Pregnancy Childbirth, 2019. **19**(1): p. 440.
23. Diarra, S.S., et al., *Factors Associated with Intermittent Preventive Treatment of Malaria During Pregnancy in Mali.* J Parasitol, 2019. **105**(2): p. 299-302.
24. Adewole, A.O., et al., *Determinants of intermittent preventive treatment of malaria among women attending antenatal clinics in primary health care centers in Ogbomoso, Oyo State, Nigeria.* Pan Afr Med J, 2019. **33**: p. 101.
25. Nkunzimana, E. and M.S. Babale, *Knowledge and Utilisation of Intermittent Preventive Treatment of Malaria among Pregnant Women in Muramvya Health District, Burundi, 2018.* East Afr Health Res J, 2020. **4**(1): p. 81-91.
26. Makenge, G., *The predictors of uptake of optimal doses of sulphadoxine pyrimethamine for intermittent preventive therapy of malaria during pregnancy among postpartum women in Tandahimba district, Tanzania*. 2019, Muhimbili University of Health and Allied Sciences.
27. Addai-Mensah, O., et al., *Regular Antenatal Attendance and Education Influence the Uptake of Intermittent Preventive Treatment of Malaria in Pregnancy: A Cross-Sectional Study at the University Hospital, Kumasi, Ghana.* Journal of Tropical Medicine, 2018. **2018**: p. 5019215.
28. Nkoka, O., T.-W. Chuang, and Y.-H. Chen, *Association between timing and number of antenatal care visits on uptake of intermittent preventive treatment for malaria during pregnancy among Malawian women.* Malaria Journal, 2018. **17**(1): p. 211.
29. Toure, O.A., et al., *Coverage and efficacy of intermittent preventive treatment with sulphadoxine pyrimethamine against malaria in pregnancy in Côte d'Ivoire five years after its implementation.* Parasites & Vectors, 2014. **7**(1): p. 495.
30. Stephen, A.A., et al., *Factors influencing utilization of intermittent preventive treatment for pregnancy in the Gushegu district, Ghana, 2013.* Pan Afr Med J, 2016. **25**(Suppl 1): p. 4.

31. Muhammad, H.U., et al., *Malaria prevention practices and delivery outcome: a cross sectional study of pregnant women attending a tertiary hospital in northeastern Nigeria*. Malaria Journal, 2016. **15**(1): p. 326.
32. Doku, D.T., M.M. Zankawah, and A.B. Adu-Gyamfi, *Factors influencing dropout rate of intermittent preventive treatment of malaria during pregnancy*. BMC Res Notes, 2016. **9**(1): p. 460.
33. Rassi, C., et al., *Assessing supply-side barriers to uptake of intermittent preventive treatment for malaria in pregnancy: a qualitative study and document and record review in two regions of Uganda*. Malaria journal, 2016. **15**(1): p. 1-16.
34. Klein, M.C., et al., *"There is no free here, you have to pay": actual and perceived costs as barriers to intermittent preventive treatment of malaria in pregnancy in Mali*. Malaria Journal, 2016. **15**(1): p. 158.
35. Kamuhabwa, A.A., R. Gordian, and R.F. Mutagonda, *Implementation of co-trimoxazole preventive therapy policy for malaria in HIV-infected pregnant women in the public health facilities in Tanzania*. Drug Healthc Patient Saf, 2016. **8**: p. 91-100.
36. Diala, C.C., et al., *Perceptions of intermittent preventive treatment of malaria in pregnancy (IPTp) and barriers to adherence in Nasarawa and Cross River States in Nigeria*. Malar J, 2013. **12**: p. 342.
37. Maheu-Giroux, M. and M.C. Castro, *Factors affecting providers' delivery of intermittent preventive treatment for malaria in pregnancy: a five-country analysis of national service provision assessment surveys*. Malaria Journal, 2014. **13**(1): p. 440.
38. Kyomuhendo, P., *Factors Affecting Regular Uptake Of Antenatal Services Among Pregnant Mothers In Their 3rdtrimester At Kisugu Health Center III*. 2018, International Health Sciences University.
39. Diengou, N.H., et al., *Factors associated with the uptake of intermittent preventive treatment of malaria in pregnancy in the Bamenda health districts, Cameroon*. Pan Afr Med J, 2020. **35**: p. 42.
40. Sangho, O., et al., *Determinants of intermittent preventive treatment with sulfadoxine–pyrimethamine in pregnant women (IPTp-SP) in Mali, a household survey*. Malaria Journal, 2021. **20**(1): p. 231.
41. Mutanyi, J.A., et al., *Determinants of the uptake of intermittent preventive treatment of malaria in pregnancy with sulphadoxine pyrimethamine in Sabatia Sub County, Western Kenya*. Infectious Diseases of Poverty, 2021. **10**(1): p. 106.
42. Buh, A., et al., *Prevalence and Associated Factors of Taking Intermittent Preventive Treatment in Pregnancy in Sierra Leone*. Tropical Medicine and Infectious Disease, 2019. **4**(1): p. 32.
43. Vincenz, C., et al., *Risk factors for placental malaria, sulfadoxine-pyrimethamine doses, and birth outcomes in a rural to urban prospective cohort study on the Bandiagara Escarpment and Bamako, Mali*. Malar J, 2022. **21**(1): p. 110.
44. Desai, M., et al., *Epidemiology and burden of malaria in pregnancy*. The Lancet infectious diseases, 2007. **7**(2): p. 93-104.
