# Supplementary material for: Level of and factors associated with optimal uptake of intermittent preventive treatment for malaria in pregnancy at private-not-for-profit health facilities in Kasese district
Source: PLOS Glob Public Health. 2024 Apr 3;4(4):e0002622. doi: 10.1371/journal.pgph.0002622 (PMC10990232; doi:10.1371/journal.pgph.0002622)
Supplement: Attachment — Submitted filename: Review Report - Number PGPH-D-23-01526.pdf [file pgph.0002622.s003.pdf]

# Manuscript Number: PGPH-D-23-01526

Thank you for the opportunity to review this manuscript, a cross-sectional study involving 396 post-partum mothers in the postnatal wards of 8 PNFP health facilities in Kasese district. The authors found that over half of the sample had received the recommended optimum number of IPTp-SP against Malaria in pregnancy, a coverage comparable primarily to what has been observed in other studies, even though still below the national and international target. Malaria, especially among pregnant women, remains a troubling public health issue, placing a high burden on the national budgets of endemic countries and negating international efforts. Thus, I commend the authors for adding to the evidence of poor adherence to the interventions against malaria, especially among vulnerable populations, such as pregnant women.

Nevertheless, there are some issues that authors need to address to move the manuscript forward.

## Major observations

**Line 68 - Methods: general comment**:
It is not clear what variables/indicators were collected from the selected women. The authors need to provide a Table of the specific variables/data collected from the women and how these were measured. For example, how was the question 'number of SP doses taken' administered? Was it mainly self-reported by the respondents? Did the authors review any other ANC records aside from HIV status (if so, at what point in time during the data collection was that done)?

This information is significant to appreciate better the commendable uptake of SP recorded in this study. For instance, a monthly dose of SP is comprised of three tablets. So, the probability that some mothers misreported a single dose as 3 doses (by count of tablets) is very high, and this could have inflated the responses to the question (since it was mainly respondent reported), particularly since the respondents were shown the SP tablets.

**Line 79-87:** It is commendable that the authors provided the number of health facilities in the study area and how many they selected (8/15 PNFP). They did well by focusing on the private, not-for-profit health facilities, which often escape the radar of many researchers, likely because some of these facilities want to be left alone to run things their way due to their "private" status.'

That notwithstanding, it is essential to contextualize the selection procedure to clarify how selection bias was controlled. Thus, the authors should clarify the following questions:
1. Why did they select 8 of the PNFP facilities, not more or less, and how did they arrive at the decision to select that number?

2. As these facilities were sampled 'purposively', it is important to have a pictorial idea of where each selected facility is located to know how spread out or close up they are relative to one another and their coverage area. Thus, it would be advantageous for the authors to provide a district map highlighting the estimated (or better specific) locations of each of the selected facilities relative to the geographical layout of the district.

3. Roughly, how many postnatal women attended/accessed each of the selected facilities relative to the estimated number of expected pregnancies per annum in the district in the previous year and in

the current year of this study? It may be helpful to provide a Table containing the names of the selected facilities, their respective ANC populations (for the previous and current year of study, respectively), and the exact number (proportion) of postnatal women they selected from each facility.

**Line 152 -**Table 1:
1. The categories of Marital status add up to >100%. Please revise.

2. The authors indicated that 269 (67.9%) of their sample had said they were "married". However, those living with their husbands are more (305) compared to those (269) married: "mothers living with their **husbands**" = 396 (305 yes+91 no). Thus, one wonders if the authors had different applicable definitions for "married" and "husbands". Logically, the question of 'mothers living with their husbands' should apply to only the 269 married women. Only "married women should be asked about their "husbands" and could be living with them ("husbands"), unless the definition of husband was applied differently to different women in this study (which will be problematic).

Therefore, the output of 305 (77.1%) yes for mothers living with their husbands looks erroneous, as it is greater than the proportion of "married" women (67.9%).

Thus, there appears to be a possibility of misclassification, and this could influence the analytical statistics (modelling association) performed.

Lines 156-160: The subsequent Table (I presume should be Table 2) indicates that '185(46.7) mothers made 4 or more 4 visits'.

Lines 161-162: Previously, the authors said, "approximately a half 211 (53.3) of the mothers attended more at least 4 visits". Now, they say "Overall, 211/396 (53.3%) attended ANC care for less than 4 visits...". The two statements contradict each other and should be clarified.

Since the total sample is less than 422, it is unclear how 211 could attend 4 or more visits and how another 211 could attend less than 4 visits.

Lines 164-168: How exactly the "Knowledge of mothers on Malaria in pregnancy..." was measured is unclear. What questions were used, and how were these obtained and measured?

How did the authors classify "Not adequate" versus "adequate" knowledge - what cut-off point or benchmark was used, and how was that derived?

**Line 182**: The authors titled this section as "level of optimal uptake of three or more doses of IPTp", but they say "...204 mothers took =>3doses of IPTp-SP or =95% adherence to cotrimoxazole giving an overall prevalence of 51.5% (46.6-56.4).". Whether the authors are referring to IPTp, cotrimoxazole, or both drugs concerning the 95% adherence is very confusing. By "overall prevalence", do the authors mean to say they combined the prevalence of SP and cotrimoxazole?

The authors are advised to present findings on the main objective of the study - uptake of IPTp-SP, instead of muddying it with other therapies.

Uptake of SP across SSA is generally low, even in settings where such essential ANC services are freely provided.
Considering the following: the authors indicated that,
1. Close to half of the participants in this study 'lived >5Km from their health facilities'.
2. All participants 'had to pay some amount out-of-pocket to access ANC services',
3. Over half (53.3%) made less than the required minimum of 4 ANC visits, and
4. About 40% did not receive SP as DOT.

Therefore, it sounds fascinating that over "95% mothers (likely they mean to say 52%) took =>3 or more doses of IPTp-SP". Compared to the available literature on IPTp-SP uptake, the over 95% uptake recorded in this study looks exciting inasmuch as it needs some clarifications:
1. The authors should clarify how the variable/question of 'number of SP doses taken so far' was administered/measured. Please refer to the comment in the methods section.

## Minor observations

Line 5: Please check the journal's standard to be sure the title is required at this stage.

General comment on Abstract:
Conventionally, abbreviations are not commonly used in the abstracts of scientific papers. Please check the journal's standard to ensure this is an exception. Otherwise, it is best to provide the complete definitions instead, albeit the word limitation imposed by the journal - "not more than 300".

**Abstract**
Line 15: "sub optimal" is better written as a word - 'suboptimal' or hyphenated - 'sub-optimal' instead.

Line 17: The sentence appears to be missing an article - 'the' and the preposition - 'of'. Please check and consider revising as follows: '*...the level of and factors associated with **the** uptake **of** IPTp...*'

**Line 17:** Note that "not for profit" is better written as a hyphenated expression - 'not-for-profit'

Line 20: What do "HC IVs" and "HC IIIs" mean?

Line 22: The word "semi-**S**tructured" does not need to be capitalized unless there is a particular context-wise significance; in that case, this should be clarified.

Line 25: Please check and indicate clearly if "(46.6-56.4)" is the confidence interval (CI) of the "51.5%" uptake, as I presume.

Lines 29-32 (abstract's Conclusion): I presume that the authors intend a structured abstract. If so, please adjust the conclusion to be a separate paragraph to comply with a structured abstract format.

Lines 34-36 (Keywords): The keywords appear as 'list of abbreviations'.

Also, use punctuation to distinguish each keyword from the others clearly.

**Background**
Line 38: The introductory sentence appears to be missing a full stop. This should be fixed to break the sentence after the first citation "1".

Lines 30-40: Please consider revising as follows - '*Sub-Saharan Africa continuously has the highest incidence of malaria among the risky populations, translating to 95% of the global burden of malaria cases...*'

**Lines 42-43**: Please provide the appropriate citation(s) for the information.

Since the information in the introductory paragraph is essential for the topic in question, the authors need to rearrange it to highlight the logical flow and clarity of thoughts. Thus, from global - continental - country/national - sub-national, etc. E.g., information in lines 44-45 could better come after lines 38-40. For instance: "*Globally Malaria is a major public health problem with an estimated 219 million cases and 435,000 deaths [1]. Sub-Saharan Africa continuously has the highest incidence of malaria among the risky populations, translating to 95% of the global burden of malaria cases. In Africa, malaria in pregnancy poses a 3-4 times risk of miscarriage, low birth weight and increase stillbirth[5].*
In Uganda, malaria remains the highest contributor (29.1%) of outpatient cases, with 10.9 % of all deaths [41].... Placental transmission among pregnant women accounts for about 45% of malaria transmissions in Uganda's high malaria transmission settings... In response, Uganda adopted...'

**Lines 45-47**: Please consider revising as follows - '*In response, Uganda adopted the WHO recommendations [6] which call for daily cotrimoxazole for pregnant women who are HIV positive, and at least 3 doses of IPTp-SP throughout for pregnant women who are HIV negative, schedule at one dose a month, beginning from the second trimester of pregnancy*.'

Please move the citation to the end of the sentence since the remaining information about IPTp-SP is referenced from the same source.

**Lines 47-50**: Please consider revising as follows:
'*Although an analytical cross sectional study linked optimal uptake of IPTp to 73.3% reduction in malaria infections and anemia by more than half [7], optimal uptake of IPTp remains very low (14.7%) across Uganda [8] due to multiple challenges*.'

**Lines 47-48**: Please delete "...in Mbale Regional Referral Hospital among 184 mothers in labour..."

**Line 49**: Please remove the full stop between "...half" and the citation "*[7]*", since it appears that the sentence is continuous after the citation.

**Line 52:** Replace "... they still have gaps in ..." with '*there is still a challenge...*'

**Line 54:** The "D" in "Directly observed treatment" must not be capitalized. Alternatively, it is better to capitalize each word or none to ensure uniformity.

**Line 55:** To use an abbreviation for the first time without its full meaning, it should have been previously defined. E.g., private not-for-profit (PNFP).

Note that abbreviations are generally not allowed in abstracts but in the main text by convention.

**Line 57:** Please revise " without its full meaning" as '*They charge* ***for*** *some services like...*'.

Additionally, especially since the health facilities are not-for-profit, it is helpful to clarify if the charge for these services is captured by the health insurance - Uganda National Minimum Health Care Package or not. Is it charged to those without health insurance, or is it out of pocket even if one has the Uganda National Minimum Health Care Package?

Line 60: consider using 'hard-to-reach' instead of "hard to reach".

Line 84: Please end the sentence after "...was done" and resume the following sentence (beginning from "...these...") in its own merit.

Line 93: I assume the authors meant the square root of d (margin of error) instead of "d2" - "d2" is not the same as 'd-squared'.

**Lines 100-101 and 103-105: It appears that the text in these lines has a different font size than** the surrounding text. Please check (see the highlighted portion in the commented manuscript).

**Lines 117-118:** Provide appropriate citation(s) to support the 10%confounding criteria.

**Lines 122-126:** The sentence is too long and verbose. Please revise it into at least two sentences.

Line 133: Avoid using conjugations - "didn't" - in formal scientific writings.

**Line 136:** "un employed" should be written as a word, not separated. Please revise.

**Line 152 -**Table 1:
1. The categories of Marital status add up to >100%. Please revise.

Lines 156-160: Using punctuation to break the sentence into concise, readable, and clear formats would be more beneficial.

The authors may consult a professional English writer to revise the entire manuscript.

What the authors mean by "...majority 281 (71.0%) amount paid per ANC visit ..." is also unclear.

**Line 156:** Replace "approximately a half..." with 'A total of...'

Lines 162-163: Please clarify in what currency the payment of "...less than 10,000..." was made.

Lines 164-168: As suggested, please revise this and all long and winding sentences into simpler and clearer formats.

Line 165: It is unclear what the authors mean by "multi-porous" and if that could be applicable in the context of this study. Perhaps they want to say 'multi-parous' instead, as in multi-parity?

**Line 178 - page 10:** The Table numbers do not appear in a series. Please check and revise. Tables need to be clearly distinguished by different numbers.

**"Table 1"** (likely Table 2): "Who met costs for IPTp drugs(N=355)". Please, replace "met" with 'paid' instead. Also, specify the currency.

Replace "gestation age" with 'gestational age' instead.

Line 182: Delete "...eligible..."

Line 189: Please double-check the presentation of all CIs. E.g., "CPR.=084, 95%CI 0.0.68-1.02" is problematic. I presume the authors meant to write 'CPR =0.84, 95%CI: 0.68-1.02'.

Line 193: "(... **0.1.61)**"

Line 193: Replace "table 2 and 3" with 'tables 2 and 3'.

**Table 4:** Please check - the figures for ANC visits seem inconsistent throughout the manuscript. 'Table 2' indicates that about 46% made 4 or more ANC visits, while Table 4 shows 61.6%. By the way, 185 of 204 does not yield 61.6%. Perhaps I have misread the information presented.

Also note that while 121+71=192 for the sub-optimal, 90+185 is more than the optimal proportion of 204.

How was "**Malaria status of mothers in last 3 months**" measured - secondary medical record review or active laboratory investigations (RDTs + microscopy)?

How did the authors prevent or reduce the possibility of false-positive Malaria test results?

Table 4: Please see 'multi-parous' for "multi-porous".

See 'gestational age' for "gestation age".

**Lines 215-376 - Discussion:**
General observations
- the authors sub-headed the discussion, and this is unnecessary and unconventional. The format of a scientific manuscript should not be comparable to a compiled thesis. Please look at the Discussion sections of other published papers, albeit the author guide of the journal, and format accordingly.

Ideally, the recommendation paragraph should come before the paragraph on limitations.

- There appear to be inconsistencies in the in-text citations: in some cases, the authors use a full stop before the citation. In other cases, they use a comma, and sometimes, there is no punctuation. Please check and revise appropriately viz-a-viz other examples published in the journal.

- The discussion is generally too long and seemingly inflated by redundant information. The authors should consider revising it to be more concise.

**Lines 216-219**: Please delete lines 216-219, as the information does not add significance to the paper. A typical discussion should begin with a concise summary of the study's main findings. The authors can check other publications in the journal to serve as a guide.

**Lines 221-227:** The authors assert that 'about 6 of 10 women (60%) in the current study received the optimal number of SP doses, and they compared this finding to a Malawian study which reported 4 of 10 women optimal uptake.' This comparison is problematic for the following reasons:

1. 51.5% of optimum SP uptake is better translatable 5/10, not 6/10, because the difference is 10%, and 10% of 396 is approximately 40 more women.
2. The Malawian study reported a finding of 30.2%, which is 10% less than the authors quoted. So, one may argue that the authors made up their finding by +10% and the Malawian study by the same percentage. This adjustment (other than the reality) is not appropriate.

Consequently, the finding on optimal SP uptake in the current study is roughly 20% higher than reported in the Malawian study. Thus, the two studies are not necessarily comparable even though both are below the WHO and national targets. Therefore, it is better not to use the percentile scores as the basis of the comparison. Instead, please clarify that the basis of comparing the two studies is on the 'WHO benchmark of 93%'.

**Lines 230-232**: It is problematic to say that the moderate coverage of optimum uptake in this study is due to a subsidized payment system. No statistical evidence is presented in this study to support this claim.

The authors reported that "**281(71.0)**" - 7 out of 10 women - paid less than 10,000 (Shillings) to access the ANC services. There is no explicit guarantee that **all** the 51.9% of women who reportedly took 3 or more doses of SP were among the 71% who paid less than 10,000 (Shillings). Since the authors have not presented statistical evidence (significant positive association) between the amount of money spent on ANC services and uptake of SP in this study, it may be better to speculate the possible reasons for the optimal uptake (amidst other factors), other than the claim of 'subsidized cost'.

**Lines 233-235**:

As commented earlier, the authors say that they 'interviewed "mothers who had delivered within 72 hours..." and this approach "...might have led to this high results because this method reduced recall bias since some of them had attended ANC before, so it was easy for
them to remember the drugs they had taken." Please correct the missing concord in “... this high results...”.

This argument presents a challenge since the data on the number of SP doses taken were self-reported by respondents other than interviewer review of ANC records. This approach tends to introduce high "false-positive" responses - response bias, in that some mothers, especially the 40% who reportedly received their doses out of the recommended DOT, could claim they took the drug when the reality could be different. Thus, the optimum uptake coverage reported in this study could be less.

This needs to be mentioned in the limitation section to keep readers and decision-makers in context.

**Line 239-241:** The authors say, "In Uganda, the available **studies have** been conducted in public health facilities, and they have consistently reported low uptake of three or more IPTp doses evidenced by 14.7% in the study of determinants of uptake of malaria preventive interventions in eastern Uganda. [8]. The low uptake recorded in their study could have been due to missed opportunities, as indicated in the literature on the barriers of IPTp uptake in Uganda. [9]."

This is problematic as they cited a single study - Wafua et al., (2021), a one-time study, instead of many studies. To say that "**studies"** have consistently shown low uptake is to logically suggest that many studies have been done over a while, and the results have repeatedly revealed low uptake. Therefore, the expectation is to cite at least two studies specific to the issue.

Otherwise, please consider rephrasing the paragraph: '*An earlier and related study from the public health facilities in eastern Uganda reported low optimum uptake of IPTp-SP [8], and this was reportedly due to missed opportunities [9]*'.

**Line 250**: Replace "From this study..." with 'In this study...'#Lines 268-269: "These variations may be explained by 50.2% and 16.7% of our mothers having primary and no education respectively as the highest level of education and reside in the remote areas of the district." The last part of the statement, "... *as the highest level of education and reside in the remote areas of the district.*" seems dangling and unclear as to what exactly the authors are referring. It seems to have displaced the intended message the authors intended to communicate. Please check the sentence carefully and revise it to give a more precise meaning.

Line 270: replace "...didn't report..." with '...did not find...'

Lines 271-273: Since the authors said that they 'did not find "any significant association between residence and uptake of IPTp", it may not be necessary to highlight 'urban dwellers as possibly not being health conscious'. It makes it appear that rural dwellers are comparatively health conscious and adherent to SP, but urban dwellers are not. This could be misleading.

**Suggested explanation:**

Based on the finding on 'residential status' in this study, it suggests that rural or urban dwelling in the study area was not a significant determinant of optimum uptake of SP. Thus, SP uptake in both cases could be relatively homogeneous across rural and urban areas. This means that other possible participant and systemic factors influencing SP uptake, such as access to healthcare facilities, awareness, and acceptance of the medication, are likely similar in both settings. When there are no

substantial differences in these factors, a lack of a significant association between residence and SP uptake can result.

Additionally, the authors may consider checking who and how they were classified as "rural" or "urban" dwellers. It is possible that, by time frame, there is no distinct difference between the rural and urban categorization in the sample.

Furthermore, if more ANC visits are associated with optimum SP uptake in this study, what proportions of the women in rural and urban areas who made at least 4 ANC visits could be similar? How many rural and urban took optimum SP doses?

**Line 275**: "...didn't..." The authors should use standard language terminologies and refrain from conjugated expressions.

**Lines 277-278:** The authors said they did not find a significant association between occupation and optimum uptake of SP, unlike a Nigerian study, and that "the difference could be due to their large sample size and their inclusion criteria was not strict, it included both pregnant and post-partum mother."

Since the difference in sample size can influence the strength and direction of association, the issue of including pregnant women sounds unjustifiable in this case. Pregnancy is not an occupation but a temporal state of being. So, it is unclear how including pregnant women could influence the relationship between occupation and optimum uptake of SP.

Instead, it could be due to equal access (or inaccess) to SP across all occupation categories, e.g., knowledge level and or acceptability of the therapy. Thus, the larger sample in the Nigerian study was likely drawn from more expansive geographic areas and may contain significant participant characteristics variations compared to the current study sample from a few shared facilities in one district.

**Lines 284-286:**
Based on the authors' findings and argument that 'majority of the women in this study were married, married women had 35% likelihood taking optimum SP due to spousal support (because of reminders and socioeconomic advantage over single women), it pretty beats logic that
'living with the husbands was not associated with optimum uptake of SP.'
Could the authors explain this finding?

Line 291: Please avoid the use of linguistic jargon. Instead, use simplistic and scientifically acceptable expressions, e.g., 'this finding supports, is comparable, is similar to, etc.'

Line 293: Please use " consistent with..." instead of "consistent to..."

Additionally, avoid the repetition of data in the discussion section as much as possible. e.g., "aOR = 1.55 (95% CI = 1.34–1.80)"

**Lines 297-298:** "As reported in this study, it was also found that DOT practice and health talk at the facility confirmed by ANC clients as satisfactory did not positively influence uptake of IPTp [30]."

Could the authors clarify where the data supporting the 'satisfactory confirmation of DOT practice and health talk' are presented in this study?

Line 300: Avoid using conjugated expressions, as this is a scientific manuscript intended for publication. Please, check the entire script to ensure that similar instances are revised appropriately.

The word "adhered" should be in the present. You cannot consecutively use two past tense verbs - "...didn't adhered...".

Line 302: "...town, Therefore, there...". Please use proper punctuation and adhere to grammatical rules. A capital-lettered word cannot follow a comma unless that word is a standard known contextual term or you are beginning a fresh sentence.

Line 305: Perhaps the authors intended to use 'were' instead of "had". Please revise.

Line 306: Do the authors mean 49% instead (I assume the reference is to Table 4)?

As attendance was measured as a variable, it is better to use 'attendance' instead of "attendances" and adjust the follow-up verb in concord. E.g., it would be inappropriate to say, '*majority of the mothers had low knowledges*'.

Line 311: Since 70% did not have to pay for their SP, it would be interesting to know if those who paid for the SP drugs paid at the health facilities they went for ANC or they bought from the open market.

**Lines 315-316 (in reference to line 311**): the authors say that 'mothers who paid for their SP drugs had 26% less likely to complete their recommended SP therapy'. However, in lines 315-316, they said that the "...significant association can be due to that fact that when mothers buy drugs for themselves, they attach more value to the drugs and hence increasing uptake". This is contradictory and needs to be clarified since '26% less likelihood rather suggests that mothers who paid for SP are demotivated to completing their recommended SP therapy, and therefore does not translate into optimum uptake.'

**Lines 326-333:** The two paragraphs appear irrelevant, considering the focus of this study. The authors may consider keeping the related information in the results section, but they do not necessarily need to discuss it. So, consider deleting the two paragraphs.

In any case, 26.3% of malaria cases should not be expected to be positively associated with SP uptake. Instead, it could indicate the outcome of poor adherence to the preventive measures - there could have been a negative association, if any.

Line 326: Do the authors mean 'with regards to' instead of "In regard to"?

Lines 344-346: There is redundancy in the text of these lines. Please revise.

**Line 349:** "The current study did not find an association with knowledge of IPTp...". "association" naturally implies a relationship between two or more things/ideas. So, the authors should revise the sentence to ensure completeness. E.g., '*an association between X (maternal knowledge of...) and Y (optimum uptake of IPTp-SP)*'.

Line 349: "...contrary to this other...". Please revise to read 'contrary to another...' or 'contrary to a study...'

Lines 351-353: There appear to be two sentences with missing punctuation(s). Please check.
Also, note "...not for profit..." instead of 'not-for-profit', and the missing concord in the use of "...this none coherence findings".

Line 358: Please check citation and punctuation style in "...study. [43]."
Line 359 (and across the manuscript): The authors have repeatedly used the term "prime-gravida" or "prime gravida", but I think what they mean is 'primigravida' instead. Otherwise, it is unclear what "prime gravida" means. Also, check "...compared 22.7%..."

**Line 360:** "primagravida" does not fit the definition "...first-time mothers..." Please check and revise or clarify. Likely, the authors mean 'primigravida' instead.

Line 364: Please check the improper use of punctuation(s) in "...IPTp.however,..."

Line 365: Please check improper grammar in "... a study of among post-partum women..."

Lines 368-370: I commend the authors for recognizing and admitting the possible response bias in their sampling approach.

Lines 370-375: Lines 370-372: The statement beginning "Since the study was targeting post-partum mothers, ... visits leading to bias," contradicts the authors' previous statement (see lines 233-235): that '*interviewing mother 72 hours post-delivery ensured good recall of drugs taken and prevented recall bias*'. Please clarify.

Line 370: 'Response bias' is a better expression.
Line 372: Please check punctuations in "...bias, However, ..."

**Conclusion**
Lines 378-379: As commented earlier, the statement "The study has established that about a half of the pregnant mothers completed =>3 doses of IPTp-SP or => 95% adherence to Ctx in private not for profit (*use not-for-profit*) health facilities in Kasese." sounds very confusing and needs to be clarified.

1. It is unclear if the authors meant to highlight the findings on their study objective or all drugs taken at ANC. The conclusion should focus on SP uptake other than cotrimoxazol.

**Line 384:** The authors said that '...based on the results, ... mothers buying IPTp drugs by themselves were significantly associated with optimal uptake of IPTp'. They also said their study 'did not find an association (between knowledge of malaria in pregnancy) and IPTp-SP.' However, they recommended that "... health education on malaria in pregnancy and IPTp as well as removing charges on ANC services may be important in increasing the uptake of IPTp in this district...".

**Clarifications:**
1. Why do the authors recommend the removal of charges if their finding says self-buying (instead of free supply) of IPTp improves its uptake? This suggests that the authors may need to re-examine the interpretation of their finding on the association between payment for ANC services and uptake of IPTp-SP.

2. Why do the authors recommend health education on malaria in pregnancy if knowledge of malaria in their study showed no association with optimum IPTp uptake?

**Line 386:** The authors may consider revising the last paragraph of the conclusion section to include a sentence or two on recommendation instead of a separate section.

**Line 419: Declaration**
The authors may want to carefully evaluate the content and logical flow of the manuscript as it seems there may be substantial direct copy-and-paste from a "dissertation" (see line 420). Please check the suggested declaration statement provided by the journal of interest and revise it appropriately.

Clarify lines 437 versus line 429.

**References**
1. Please ensure that **all** references are reflected correctly in the text and vice versa. Please use the appropriate settings in the referencing software.

2. Use the appropriate referencing style, e.g., why is it necessary to abbreviate the author's name - WHO, and still write the full meaning - World Health Organization, but others (MOH) are left abbreviated? Names of such references should be written in full in the references section.

3. Where applicable, provide each reference's DOI or active URLs for easy verification.
