# Supplementary material for: Level of and factors associated with optimal uptake of intermittent preventive treatment for malaria in pregnancy at private-not-for-profit health facilities in Kasese district
Source: PLOS Glob Public Health. 2024 Apr 3;4(4):e0002622. doi: 10.1371/journal.pgph.0002622 (PMC10990232; doi:10.1371/journal.pgph.0002622)
Supplement: Attachment — Submitted filename: Response to Reviewers.docx [file pgph.0002622.s004.docx]

Mutoro Julius

Student, Clinical epidemiology Unit

Makerere University college of Health sciences

Email. juliusmutoro@yahoo.com

Tel: 0703679844

Date: 18/10/2023

**To: PLOs**

**Reviewer**

Dear,

**RE: RESPONSE TO ISSUES HIGHLIGHTED BY REVIEWERS OF MY MANUSCRIPT**

Receive the responses to the above dissertation titled***: Level and factors associated with optimal uptake of intermittent preventive treatment for malaria in pregnancy at private not for profit health facilities in Kasese district.***

This manuscript was submitted to PLOS in August 2023 and I received the comments from the reviewers by 26^th^ September 2023. Below is a table showing the responses to the issues that were raised.

| **s/n** | **Comment from Reviewer** | **Response** | **Reference in clean document** |
| --- | --- | --- | --- |
| 1. | Authors  Line 5: Please check the journal's standard to be sure the title is required at this stage. | The journal guidelines do not require titles. The title professor has been removed | **Line 4** |
| 2. | Abstract:  Conventionally, abbreviations are not commonly used in the abstracts of scientific papers. Please check the journal's standard to be sure if this is an exception. Otherwise, it is best to provide the complete definitions instead, albeit the word limitation imposed by the journal - "not more than 300.  Line 15: "sub optimal" is better written as a word - 'suboptimal' or hyphenated - 'sub-optimal' instead.  Line 17: The sentence appears to be missing an article - 'the' and the preposition - 'of'. Please check and consider revising as follows:  Line 17: Note that "not for profit" is better written as a hyphenated expression - 'not-for-profit'  Line 20: What do "HC IVs" and "HC IIIs" mean, respectively?  Line 25: Please check and indicate clearly if "(46.6-56.4)" is the confidence interval (CI) of the "51.5%" uptake, as I presume.  Lines 29-32 (abstract's Conclusion): I presume that the authors intend a structured abstract. If so, please adjust the conclusion as as a separate paragraph to comply with the format of a structured abstract.  Lines 34-36 (Key words): It appears the key words are written as 'list of abbreviations'. Also make use of punctuations to clearly demarcate each key word from the other. | The abbreviations have been defined in the abstract, However, the ones with many words like IPTp has not been avoided throughout the abstract because of the word limitations.  The word sub-optimal has been separated with a hypha  The sentence has been correctly revised to level of and factors associated with”  The word private not for profit has been hyphenated to Private-not-for-Profit  HC IIIs and HCIVs means Health centre III and Health centre IV levels of health service delivery in the Ugandan context. The word HC has been written in full as Health centre  Line 24: "(46.6-56.4)" is the confidence interval (CI) of the "51.5%" uptake. It has been correctly written as 51.5% CI= (46.6-56.4)  A paragraph has been created  The abbreviations have been removed and punctuation has been done. | Abstract Line 13-30  Line 14  Line 15  Line 16  Line 19  Line 24  Line 29  Line 32 |
|  | **Introduction**  Line 38: It appears that the introductory sentence is missing a full stop. This should be fixed to break the sentence just after the first citation "1".  Lines 30-40: Please consider revising as follows - 'Sub-Saharan Africa continuously has the highest incidence of malaria among the risky populations, translating to 95% of the global burden of malaria cases...'  Lines 42-43: Please provide the appropriate citation(s) for the information.  Inasmuch as the information in the introductory paragraph is essential for the topic in question, the authors need to rearrange them to highlight the logical flow and clarity of thoughts. Thus, from global - continental - country/national - sub-national, etc. E.g., information in lines 44-45 could better come after lines 38-40.  Lines 45-47: Please consider revising as follows - 'In response, Uganda adopted the WHO recommendations [6] which call for daily cotrimoxazole for pregnant women who are HIV positive, and at least 3 doses of IPTp-SP throughout for pregnant women who are HIV negative, schedule at one dose a month, beginning from second trimester of pregnancy.'. Please move the citation to the end of the sentence since the remaining information about IPTp-SP is referenced from the same source.  Lines 47-50: Please consider revising as follows:  'Although an analytical cross sectional study linked optimal uptake of IPTp to 73.3% reduction in malaria infections and anemia by more than half [7], optimal uptake of IPTp remains very low (14.7%) across Uganda [8] due to multiple challenges.'  Lines 47-48: Please delete "...in Mbale Regional Referral Hospital among 184 mothers in labour..."  Line 52: Replace "... they still have gaps in ..." with 'there is still a challenge...'  Line 54: The "D" in "Directly observed treatment" does not have to be capitalized. Alternatively, it is better to capitalize each word or none at at all to ensure uniformity.  Line 55: To use an abbreviation for the first time, without its full meaning, it should have been previously defined. E.g., private not-for-profit (PNFP).  Line 57: Please revise " without its full meaning," as 'They charge for some services like...'.  Additionally, especially that the health facilities are not-for-profit, it is helpful to clarify if the charge for these service is captured by the health insurance - Uganda National Minimum Health Care Package or not. Is it charged to those without any health insurance, or is it out of pocket even if one has the Uganda National Minimum Health Care Package?  Line 60: consider using 'hard-to-reach' instead of "hard to reach". | A full stop has been added to break the sentence  The statement has been revised  The citation has been inserted  The statements have been re-aligned according to the guidance provided  This statement has been revised accordingly  The citation has been moved to the end  The line has been replaced as guided  The statement has been deleted  I have capitalized each word  The abbreviation PNFP has been written full and then abbreviated since its appearing for the first time in the body of the document.  In Uganda, we don’t have community insurance and at private-not-for-profit health facilities, people pay user fees and this fee is extended to even essential services like ANC and deliveries and yet pubic facilities are offering them for free. The mothers who access ANC at PNFP facilities pay for these services because they are the ones that are very close to them in the mountainous areas.  However, I have inserted a clarification on that matter  The word has been written as hard-to-reach | Paragraph 1 line 38  Line 38  The citation has been added on line 44  From line 40 to 45  Line 44 to 47  Line 47  Line 47 to 49  Line 54  Line 55 page 3  Line 57 to 60 page 3  Line 62 |
| 3. | **Study design, participant enrollment, and data collection**  1.That notwithstanding, it is important to contextualize the selection procedure to make it even clearer to understand how selection bias was controlled. Thus, the authors should clarify the following questions:  1. Why did they select 8 of the PNFP facilities not more or less, and how did they arrive at the decision to select that number?   2. As these facilities were sampled 'purposively', it is important to have a pictorial idea of where each selected facility is located, to know how spread out or close up they are relative to one another and area of coverage. Thus, it would be advantageous that the authors provide a district map highlighting the estimated (or better specific) locations of each of the selected facility relative to the geographical layout of the district. 3. Roughly, how many postnatal women attended/accessed each of the selected facilities, relative to the estimated number of expected pregnancies per annum in the district in the previous, and in the current year of this study? It may be helpful to provide a Table containing names of the selected facilities, their respective ANC populations (for the previous and current year of study, respectively), and the exact number (proportion) of postnatal women they selected from each facility. | The HC IIs were not selected since they don’t deliver mothers and our study targeted post-partum mothers. The use of random selection for the HC IIIs controlled for selection bias. Purposive sampling was done for selecting the Hospital and the two HCIVs because they were the only highest levels of service delivery amongst all the PNFPs in the district and this makes them contribute the largest participants for the study. In addition, the three purposively selected health facilities are located in different health sub districts.  The map of the district (Fig1) showing the location of the PNFP health facilities has been provided as an attachment  More clarification has been given on proportionate sampling of the respondents and the table has been inserted. Refer to table 1. | Line 88 to 91  Fig 1 at 373 page 21  Line 99 table 1 |
| 3. | **Sample size and power calculations.**  Line 93: I assume the authors meant the square root of d (margin of error) instead of "d2" - "d2" is not the same as 'd-squared'. | In this formula, the Author d is squared. | Line 102 |
| 4. | **Statistical analysis.**  Lines 100-101 and lines 103-105: It appears that the text in these lines have a different font size compared to the surrounding text. Please check (see highlighted portion in commented manuscript). | The font has been adjusted from 11 to 12. | Line 109 to 113 |
| 5. | **Ethical considerations**  **Lines122-126:** The sentence is too long and verbose. Please revise into at least two sentences. | The statement have been revised and punctuated well. | Line 132 to 138 on page 7 |
| 6. | **Results**  Line 133: Avoid the use of conjugations - "didn't" - in formal scientific writings.  **Line 136:** "un employed" should be written as a word, not separated. Please revise.  **Line 152 -**Table 1:   1. The categories of Marital status add up to >100%. Please revise.   The authors indicated that 269 (67.9%) of their sample had said they were "married". However, those living with their husbands are more (305) than those (269) married: "mothers living with their **husbands**" = 396 (305 yes+91 no). Thus, one wonders if the authors had different applicable definitions for the terms "married" and "husbands". Logically, the question of 'mothers living with their husbands' should be applicable to only the 269 married women. Only "married women should be asked about their "husbands" and could be living with them ("husbands"), unless the definition of husband was applied differently to different women in this study (which will be problematic). Therefore, the output of 305 (77.1%) yes for mothers living with their husbands looks erroneous, as it is greater than the proportion of "married" women (67.9%). Thus, there appears a possibility of misclassification, and this could have an influence on the analytical statistics (modeling association) performed.  Lines 156-160: It would more useful to apply punctuations to break up the sentence into concise, readable and clear formats. The authors may consider consulting a professional English language writer to revise the entire manuscript.  It is also not clear what the authors mean by "...majority 281 (71.0%) amount paid per ANC visit ...".Meanwhile the subsequent Table (I presume should be Table 2) indicates that '185(46.7) mothers made 4 or more 4 visits'.  Lines 161-162: Previously, the authors said "approximately a half 211 (53.3) of the mothers attended more at least 4 visits". Now, they say "Overall, 211/396 (53.3%) attended ANC care for less than 4 visits...". The two statements contradicts and should be clarified.  Since the total sample is less than 422, it is not clear how 211 could attend 4 or more visits and another 211 attended less than 4 visits.  **Line 178 - page 10:** It appears that the Table numbers are not in series. Please check and revise. Tables need to be clearly distinguished by different numbers.  Please replace "met" with 'paid' instead. Also specify the currency.  Replace "gestation age" with 'gestational age' instead.  **Line 182**: The authors titled this section as "level of optimal uptake of three or more doses of IPTp", but they say "...204 mothers took =>3doses of IPTp-SP or =95% adherence to cotrimoxazole giving an overall prevalence of 51.5% (46.6-56.4).". It is very confusing whether the authors are referring to IPTp, cotrimoxazol, or both drugs with regards to the 95% adherence. By "overall prevalence", do the authors a mean to say they combined the prevalence of SP and cotrimoxazol ? The authors are advised to present findings on the main objective of the study - uptake of IPTp-SP, instead of muddying it with other therapies.  **Table 4:** Please check - the figures for ANC visits seem inconsistent throughout the manuscript. 'Table 2' indicates about 46% made 4 or more ANC visits, while Table 4 shows 61.6%. By the way, 185 of 204 does not yield 61.6%. Perhaps I have misread the information presented. Also note that while 121+71=192 for the sub-optimal, 90+185 is more than the optimal proportion of 204.  How was "**Malaria status of mothers in last 3 months**" measured - secondary medical record review or active laboratory investigations (RDTs+microscopy)? How did the authors prevent or reduce the possibility false-positive Malaria test results? | The conjugation didn’t have been adjusted to “did not”  The word un employed has also been written as “unemployed”  When I went back to the data set, I found out that widows were recorded and yet they were not analyzed so I removed the raw for widows from the table.  I realized that the divorced and single mothers were asked whether they lived with their husbands and this question was not applicable to them. We were supposed to ask only the mothers who were married. I have then captured the responses from the divorced and single mothers as “Not applicable”. On re-analysis, the variable of living with husband was significant at bivariate analysis with a Pvalue 0.003 less than the cut of 0.2. (Table 2) However, at multivariate analysis, the variable did not become significant and it was therefore dropped from the model. Table 4 has been updated to include the affected variable.  The sentences have been punctuated according to the guidance provided  The paragraph has been deleted since it was repeated in the previous statement  Table numbers have been revised The word met has been replaced with paid  The word gestation age has been replaced with gestational age  In this context, the authors considered optimal uptake of IPTp to be HIV negative mothers who took 3 or more doses of IPTp-SP plus HIV positive mothers who had adherence of >95% to cotrimoxazole. Use of Cotrimoxazole for HIV positive mothers was taken as a prophylaxis for malaria among the HIV positive mother in care.  This has been updated  Mothers were asked if they had episodes of malaria in the last three months and their responses were captured. | Line 143 page 7  Line 146 page 7  Line 152, table 2 on page 8  Line 152, table 2 on page 8  Line 155 to 160  Table 3 page 9  Table 3 page 9  Table 5 page 12 |
| 7. | **Discussion**  Lines 216-219: Please delete lines 216-219 as the information does not add any significance to the paper. A typical discussion should begin with a concise summary of the main findings of the study. The authors can check other publications in the journal to serve as guide.  Lines 221-227: The authors assert that 'about 6 of 10 women (60%) in the current study received optimal number of SP doses, and they compared this finding to a Malawian study which reported 4 of 10 women optimal uptake.'  This comparison is problematic for the following reasons:  1. 51.5% of optimum SP uptake is better translatable 5/10, not 6/10, because the difference is 10%, and 10% of 396 is approximately 40 more women.  2. The Malawian study reported a finding of 30.2%, which is 10% less than the authors quoted. So, the authors literally made up their finding by +10% and the Malawian study by same percentage. This is not appropriate.  Consequently, the finding on optimal SP uptake in the current study is roughly 20% higher than reported in the Malawian study. Thus, the two studies are not necessarily comparable even though both are below the WHO and national targets. It is therefore better not to use the percentile scores as the basis of the comparison. Instead, please clarify that the basis of comparing the two studies is on the 'WHO benchmark of 93%'.  **Lines 230-232**: Again, it is problematic to say that the moderate coverage of optimum uptake in this study is due to subsidized payment system. There is no statistical evidence presented in this study to support this claim. The authors reported that "**281(71.0)**" - 7 out of 10 women - paid less than 10,000 (Shillings) to access the ANC services. There is no clear guarantee that **all** the 51.9% women who reportedly took 3 or more doses of SP were among the 71% who paid less than 10,000 (Shillings). Since the authors have not presented statistical evidence (significant positive association) between the amount of money spent on ANC services and uptake of SP in this study, it may be better to speculate the possible reasons for the optimal uptake (amidst other factors), other than the claim of 'subsidized cost'.  **Lines 233-235**:  As commented earlier, the authors say that, they 'interviewed "mothers who had delivered within 72 hours..." and this approach "...might have led to this high results because this method reduced recall bias since some of them had attended ANC before, so it was easy for  them to remember the drugs they had taken."  This argument presents a challenge since the data on the number of SP doses taken were self-reported by respondents other than interviewer review of ANC records. This approach actually has the tendency of introducing high "false-positive" responses - response bias, in that, some mothers, especially the 40% who reportedly received their doses out of the recommended DOT, could claim they took the drug when the reality could be different. Thus, in reality, the optimum uptake coverage reported in this study could be less.  This needs to be mentioned in the limitation section to keep readers and decision-makers in context.  Lines 268-269: "These variations may be explained by 50.2% and 16.7% of our mothers having primary and no education respectively as the highest level of education and reside in the remote areas of the district." The last part of the statement "... *as the highest level of education and reside in the remote areas of the district.*" seems dangling and unclear as to what exactly the authors are referring. It seems to have displaced the intended message the authors intended communicating. Please check the sentence carefully and revise to give a clearer meaning.  Lines 271-273: Since the authors said, that they 'did not find "any significant association between residence and uptake of IPTp", it may not be necessary to then highlight 'urban dwellers as possibly not being health conscious'. It makes it appear as if rural dwellers are comparatively health conscious and are adherent to SP, but the urban dwellers are not. This could be misleading.  **Line 275**: "...didn't..." The authors should use standard language terminologies and refrain from conjugated expressions.  **Lines 277-278:** The authors said they did not find significant association between occupation and optimum uptake of SP unlike a Nigerian study, and that "the difference could be due to their large sample size and their inclusion criteria was not strict, it included both pregnant and post-partum mother."  **Lines 284-286:** Based on the authors finding and argument that ‘majority of the women in this study were married, married women had 35% likelihood taking optimum SP due to spousal support (because of reminders and socioeconomic advantage over single women), it pretty beats logic that 'living with the husbands was not associated with optimum uptake of SP.'. Could the authors provide an explanation for this finding?  Line 291: Please avoid the use of linguistic jargons. Instead, use simplistic and and scientifically acceptable expression e.g., 'this finding supports, is comparable, is similar to, etc.'  Line 311: Since 70% did not have to pay for their SP, it would be interesting to know if those who paid for the SP drugs, paid at the health facilities they went for ANC or they bought from the open market.  Lines 315-316: reference to line 311, the authors say that 'mothers who paid for their SP drugs had 26% less likely to complete their recommended SP therapy'. However, in lines 315-316, they said that the "...significant association can be due to that fact that when mothers buy drugs for themselves, they attach more value to the drugs and hence increasing uptake". This is contradictory and needs to be clarified, since '26% less likelihood rather suggests that mothers who paid for SP are demotivated to completing their recommended SP therapy, and therefore does not translate into optimum uptake.'  Lines 326-333: The two paragraphs appear to be irrelevant considering the focus of this study. The authors may be considering keeping the related information in the results section, but they do not necessarily need to discuss it. So, consider deleting the two paragraphs.  In any case, a 26.3% malaria cases should not be expected to be positively associated with SP uptake. Instead, it could indicate the outcome of poor adherence to the preventive measures - could have been a negative association if any at all.  Lines 344-346: There is redundancy in the text of these lines. Please revise.  Line 349: "The current study did not find an association with knowledge of IPTp..." The term "association" naturally implies a relationship between to two or more things/ideas. So, the authors should revise the sentence to ensure completeness. E.g., '*an association between X (maternal knowledge of...) and Y (optimum uptake of IPTp-SP)*'  Lines 351-353: There appear to be two sentences, but with missing punctuation(s). Please check. Also note "...not for profit..." instead of 'not-for-profit', and the missing concord in the use of "...this none coherence findings".  Line 358: Please check citation and punctuation style in "...study. [43]."  Line 360: Apparently, "primagravida" does not fit the definition "...first-time mothers..." Please check and revise or clarify. Likely you mean 'primigravida' instead. | This has been deleted as per the guidance given.  This has been revised and appropriately stated  This has been corrected to 5 in every 10 mothers  This statement has been deleted since it was not adding value  The Uganda shillings have been converted to dollar throughout the document  During data collection, we could interview the mothers and then cross check their responses with their antenatal card.  This statement has been revised to “In this study, the variations could be explained by the low education levels of the mothers at 50.2% and 16.7% for primary and no education respectively among mothers.  The discussion on residence was deleted to save more space  The conjugate was removed and the statement written as “did not”  The statement has been re-phrased well  Based on the analysis that was made, there was no significant association  The word consonance has been changed to “consistent”  The variable on where the mothers might have bought the drugs was not collected hence not included in the analysis  This has been written as “This significant association can be due to that fact sometimes the mothers fail to buy the full prescribed doses of IPTp and end up getting demotivated to complete the dose”.  The two paragraphs have been deleted  The line has been revised to “As observed among postpartum women in Tanzania's Tandahimba district [25], mothers who live with their husbands were more likely to take the recommended doses of IPTp contrary to our findings”.  The statement was revised as “Knowledge of mothers on IPTp in this study was not significantly associated with optimal uptake of IPTp contrary to the study in Cameroon and Mali respectively [40], [41]”  The statement was revised as “*In a rural to urban prospective cohort study in Mali[44]. a positive association was reported among prime gravida than in multi-gravid, contrary to our study”.*  The authors meant primigravida | Line 202  Line 200  Table 3 and 4  Line 236 page 14  Line 239 page 15  Line 240 to 245 page 15  Line 252  Line 266-268 page 16  Line 278 to 279 page 16  Line 280 to 283, page 17  Line 287 to 289, page 17 |
| 8. | Study limitations  Lines 370-375: Lines 370-372: The statement beginning "Since the study was targeting postpartum mothers, visits leading to bias," contradicts the authors previous statement (see lines 233-235): that *'interviewing mother 72 hours post-delivery ensured good recall of drugs taken and prevented recall bias'*. | This statement was deleted *“. Interviewing mothers who had delivered within 72 hours might have led to these high level of optimal uptake that was reported because this might*  *have increased recall bias”* |  |
| 9. | **Conclusion**  Lines 378-379: As commented earlier, the statement "The study has established that about a half of the pregnant mothers completed =>3 doses of IPTp-SP or => 95% adherence to Ctx in private not for profit health facilities in Kasese." sounds very confusing and needs to be clarified.  It is not clear if the authors meant to highlight the finding on their study objective or all drugs taken at ANC. Is the conclusion about SP or Ctx?  **Line 384:** The authors said that '...based on the results, ... mothers buying IPTp drugs by themselves were significantly associated with optimal uptake of IPTp'. They also said that their study 'did not find an association (between knowledge of malaria in pregnancy) and IPTp-SP.' However, they recommended that "... health education on malaria in pregnancy and IPTp as well as removing charges on ANC services may be important in increasing the uptake of IPTp in this district...". | The conclusion was on both IPTp-SP and Ctx and it has been revised  This statement has been revised | Line 300 page 17 |
| 10. | Line 386: The authors may consider revising the last paragraph of the conclusion section to include a sentence or two on recommendation instead of a separate section. | The paragraph has been revised to include the recommendations. | Line 306 to 312 page 18 |
| 11. | Clarify lines 437 versus line 429. | The statement on competing interests has been removed and remained with conflict of interest. |  |

Yours Sincerely

**Mutoro Julius**

**Master of science in Clinical Epidemiology and Biostatistics**
